# Wood Metabolomic Responses of Wild and Cultivated Grapevine to Infection with *Neofusicoccum parvum*, a Trunk Disease Pathogen

**DOI:** 10.3390/metabo10060232

**Published:** 2020-06-04

**Authors:** Clément Labois, Kim Wilhelm, Hélène Laloue, Céline Tarnus, Christophe Bertsch, Mary-Lorène Goddard, Julie Chong

**Affiliations:** 1 Laboratoire Vigne, Biotechnologies et Environnement (LVBE, EA3991), Université de Haute Alsace, 68000 Colmar, France; clement.labois@uha.fr (C.L.); helene.laloue@uha.fr (H.L.); celine.tarnus@uha.fr (C.T.); christophe.bertsch@uha.fr (C.B.); 2 Laboratoire d’Innovation Moléculaire et Applications, Université de Haute-Alsace, Université de Strasbourg, CNRS, LIMA, UMR 7042, 68093 Mulhouse cedex, France; kimwilhelm94@gmail.com

**Keywords:** grapevine, trunk diseases, wood metabolomics, *V. v.* subsp. *sylvestris*

## Abstract

Grapevine trunk diseases (GTDs), which are associated with complex of xylem-inhabiting fungi, represent one of the major threats to vineyard sustainability currently. Botryosphaeria dieback, one of the major GTDs, is associated with wood colonization by Botryosphaeriaceae fungi, especially *Neofusicoccum parvum*. We used GC-MS and HPLC-MS to compare the wood metabolomic responses of the susceptible *Vitis vinifera* subsp. *vinifera* (*V. v.* subsp. *vinifera)* and the tolerant *Vitis vinifera* subsp. *sylvestris (V. v.* subsp. *sylvestris)* after artificial inoculation with *Neofusicoccum parvum* (*N. parvum*). *N. parvum* inoculation triggered major changes in both primary and specialized metabolites in the wood. In both subspecies, infection resulted in a strong decrease in sugars (fructose, glucose, sucrose), whereas sugar alcohol content (mannitol and arabitol) was enhanced. Concerning amino acids, *N. parvum* early infection triggered a decrease in aspartic acid, serine, and asparagine, and a strong increase in alanine and β-alanine. A trend for more intense primary metabolism alteration was observed in *V. v.* subsp. *sylvestris* compared to *V. v.* subsp. *vinifera*. *N. parvum* infection also triggered major changes in stilbene and flavonoid compounds. The content in resveratrol and several resveratrol oligomers increased in the wood of both subspecies after infection. Interestingly, we found a higher induction of resveratrol oligomer (putative E-miyabenol C, vitisin C, hopeaphenol, ampelopsin C) contents after wood inoculation in *V. v.* subsp. *sylvestris*.

## 1. Introduction

Grapevine (*Vitis vinifera*) is a very economically important crop worldwide but it is susceptible to a wide range of pathogens. Currently, one of the major threats to vineyard sustainability is Grapevine trunk diseases (GTDs). The three economically major GTDs, i.e., Botryosphaeria dieback, Eutypa dieback, and esca, are widespread in wine-growing regions over the world and cause severe yield reduction. The incidence of GTDs has increased considerably over the past few decades and the economic losses of these diseases were estimated to exceed 1 billion dollars per year [1]. GTDs are considered a major threat to winegrowers since no effective plant protection strategy is available [2].

GTDs are associated with the development of a complex of xylem-inhabiting fungi, finally leading to the death of the plant. These fungi are characterized by a latent phase, where they are present in the plant as endophytes and by a pathogenic phase, where they become virulent and colonize grapevine wood [3,4]. Various symptoms associated with GTDs are: sectorial and/or central necrosis in woody tissues [5], brown stripes or cankers, leaf discolorations, and wilt of inflorescences and berries [5,6]. Concerning esca disease, it is characterized by a mild form where plant express foliar symptoms (necrosis, discolorations) and an apoplectic form with the sudden death of the vine [6].

Botryosphaeria dieback, one of the major GTDs, is associated with wood colonization by Botryosphaeriaceae fungi, especially *Neofusicoccum parvum* (*N. parvum*), which is one of the most aggressive Botryosphaeriaceae fungus associated to this disease [7]. Botryosphaeriaceae species are well-known pathogens causing dieback in apples, pine trees, and grapevines [8]. These fungi are latent pathogens in many woody hosts and are characterized by a quiescent passive life phase, followed by an active pathogenic phase [9]. Infection by *N. parvum* through pruning wounds results in internal wood cankers, foliar chlorosis and necrosis, and dieback of shoots and buds [4,10]. Since GTDs pathogens are always found in the wood but have never been isolated from the leaves of diseased plants, it has been speculated that foliar symptoms result from toxin production in the wood. Phytotoxic compounds would be translocated to leaves via the transpiration stream [2]. *N. parvum* has been shown to produce a number of different compounds belonging to different chemical classes: naphtalenones, dihydrotoluquinones, epoxylactones, dihydroisocoumarins, hydroxybenzoic acids, and fatty esters [11,12,13].

In order to better understand trunk disease development, it is necessary to study metabolic perturbations in the grapevine wood. However, to our knowledge, no metabolomic study of grapevine wood infected by Botryosphaeriaceae has been performed. Several studies have reported physiological perturbations, especially concerning carbohydrate metabolism and induction of defense responses, in different organs of vines affected by GTDs, especially leaves [6]. In esca apoplectic leaves, transcriptomic and metabolomic analyses of grapevines in the period preceding symptom appearance revealed major metabolic reprogramming, especially concerning the metabolism of sugars, amino acids, and phenylpropanoids [14]. Similarly, nuclear magnetic resonance (NMR)-based metabolomic analyses of esca-infected leaves of *V. vinifera* cv. Alvarinho demonstrated a decrease in carbohydrates associated with an increase in phenolic compounds, suggesting a rerouting from primary to specialized metabolism involved in plant defense [15]. Concerning Eutypa dieback, a transcriptomic study of leaves of *V. vinifera* cv. Cabernet-Sauvignon infected with *Eutypa lata* showed that genes whose expression was associated with a lack of symptoms in infected plants are involved in the light phase of photosynthesis. These results highlight the importance of primary metabolism for trunk disease resistance [16]. Regarding specialized metabolites, accumulation of phenolic compounds such as *trans-*caffeoyltartaric acid, *trans*-coumaroyl-tartaric acid, quercetin-3-*O*-glucoside, quercetin-3-*O*-galactoside, kaempferol-3-glucoside, and myricetin was associated with esca disease in the leaves of *V. vinifera* cv. Alvarinho [17]. In a recent study, Moret et al. [18] studied the leaf metabolome of two *V. vinifera* cv. Chardonnay clones expressing esca symptoms. They showed that metabolite fingerprint associated with esca disease differed in two clones of the same cultivar, discriminant metabolites belonging to flavonoid and phenolic compound families.

Grapevine responses in the wood have been less studied. However, analyses of modifications in green stem, cordon, and trunk during grapevine infection with esca revealed alteration of expression of genes encoding enzymes of the phenylpropanoid pathway and resveratrol contents in the three organs of diseased plants [19]. In another recent study, Rusjan et al. [20] also reported that esca infection caused a significant accumulation of gallic acid, total flavanols, stilbenes, and total analyzed phenolics in the esca decayed wood of different trunk parts of *V. vinifera* cv. Cabernet Sauvignon.

Currently, no grape variety is completely resistant to GTDs. However, epidemiologic studies in vineyards have shown that different grapevine cultivars have contrasted foliar symptom expression reflecting different tolerance levels to GTDs. These observations suggest a genetic basis for trunk disease tolerance [21,22]. In an attempt to identify novel genetic resources for resistance against trunk diseases, Guan et al. [23] conducted a broad screen to evaluate the susceptibility levels of a large panel of accessions from the Vitaceae family to Botryosphaeriaceae fungi. Several accessions of *V. v.* subsp. *sylvestris*, the ancestor of *V. v.* subsp. *vinifera*, were found more resistant to Botryosphaeriaceae artificial inoculation than the *V. vinifera* cv. Chardonnay and Gewürztraminer [23]. In an attempt to elucidate the better tolerance level to *N. parvum* in *V. v.* subsp. *sylvestris*, and to identify tolerance biomarkers, we compared the wood metabolomic responses of *V. v.* subsp. *vinifera* and *V. v.* subsp. *sylvestris* after artificial inoculation with *N. parvum*, a main pathogenic fungus associated with Botryosphaeria dieback. A representative set of 32 primary metabolites and 52 specialized metabolites were analyzed. The results give a detailed picture of metabolic alterations in the wood after colonization with *N. parvum* and a better understanding of the tolerance to GTDs observed in *V. v.* subsp. *sylvestris*.

## 2. Results

### 2.1. Changes in Primary Metabolites in Wood upon N. parvum Inoculation

**Figure 1:**
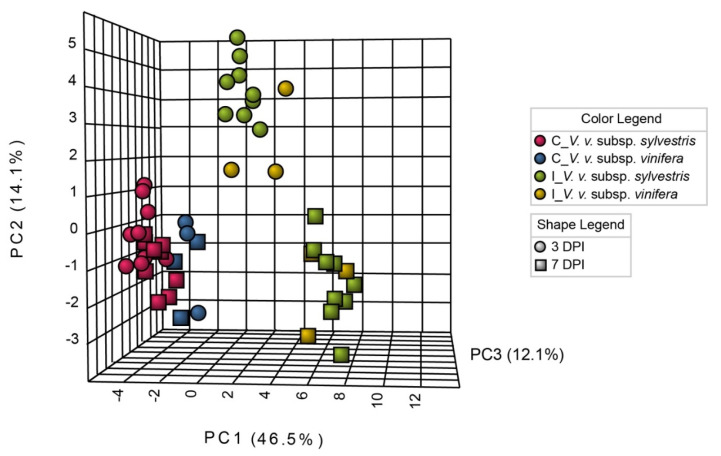
Three-dimensional principal component analysis (3D-PCA) of primary metabolite contents. PCA was performed on mean values at three and seven days post-infection (DPI) normalized to T0 non-inoculated control. Control samples (C) were mock-inoculated with potato dextrose agar (PDA), inoculated samples (I) were inoculated with *N. parvum*. Each time point is represented by a symbol (dots for three DPI and rectangles for seven DPI). The *V. v.* subsp. *sylvestris* and *V. v.* subsp. *vinifera* genotypes are represented by different colors (red for *V. v.* subsp. *sylvestris* and blue for *V. v.* subsp. *vinifera* for C samples and green for *V. v.* subsp. *sylvestris* and yellow for *V. v.* subsp. *vinifera* for I samples). The three major principal components explained 72.7% of the cumulative variance.

To determine the metabolic responses in detached grapevine canes of *V. v.* subsp. *sylvestris* and *V. v.* subsp. *vinifera* genotypes infected with *N. parvum*, we analyzed three biological replicates conducted in 2017, 2018, and 2019 using a targeted approach for primary metabolites. Each biological replicate consisted of nine *V. v.* subsp. *sylvestris* genotypes (Ke 2, Ke 34, Ke 53, Ke 83, Ke 84, Ke 95, Ke 106, Ke 119, and Hö 29) and three *V. v.* subsp. *vinifera* genotypes (Chardonnay, Gewürztraminer, and Müller-Thurgau). A total of 32 compounds including four acid sugars, five sugar alcohols, eight sugars, nine amino acids, and eight organic acids were analyzed by GC-MS (Table S1). Since T0 basal contents in metabolites differed between the three years analyzed, we expressed metabolite contents in mock (C) or *N. parvum* (I) inoculated samples as the ratio between the content in inoculated vs non-inoculated sample at T0. In order to visualize the impact of the infection on the primary metabolism in detached grapevine canes, we first performed three-dimensional principal component analysis (3D-PCA) on the I and C mean values of the three biological replicates in *V. v.* subsp. *sylvestris* and *V. v.* subsp. *vinifera* accessions (Figure 1).

Inoculation of grapevine canes with *N. parvum* resulted in dramatic changes in the content of primary metabolites. PCA analysis revealed that primary metabolite profiles strongly differed between control and inoculated samples (Figure 1). C samples from both *V. v.* subsp. *sylvestris* and *V. v.* subsp. *vinifera* genotypes were grouped together and were clearly separated from *V. v.* subsp. *vinifera* and *V. v.* subsp. *sylvestris* I samples in the PCA analysis (Figure 1). Within I samples, PCA analysis showed that primary metabolite profile significantly differed between three and seven days after inoculation, for both *V. v.* subsp. *vinifera* and *V. v.* subsp. *sylvestris*. By contrast, C samples at three and seven days were grouped together (Figure 1).

**Figure 2:**
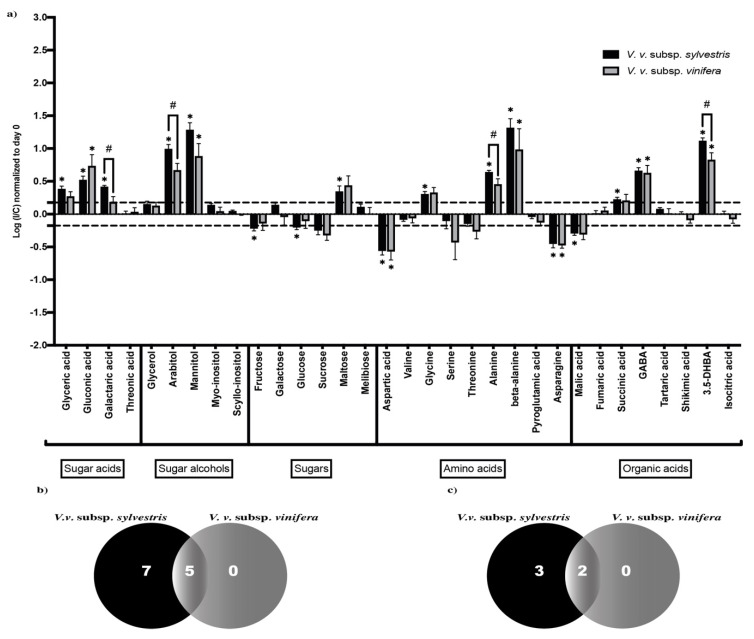
Changes in primary metabolite contents at three DPI. (**a**) Contents are expressed as the log of the ratio between content (normalized to T0) in inoculated sample (I) and content in mock-inoculated control sample (C). Metabolite contents are represented in black for *V. v.* subsp. *sylvestris* and in gray for *V. v.* subsp. *vinifera*. * means a statistically significant difference between I and C (fold change at least ± 1.5 and *p* < 0.05). # represents a (I/C) statistically significantly different between *V. v.* subsp. *sylvestris* and *V. v.* subsp. *vinifera* (*p* < 0.05). Error bar represents standard error of the mean. (**b**) Venn diagram of metabolites increasing significantly between I and C (fold change at least ± 1.5 and *p* < 0.05), in black for *V. v.* subsp. *sylvestris* and in gray for *V. v.* subsp. *vinifera*. (**c**) Venn diagram of metabolites decreasing significantly between I and C (fold change at least ± 1.5 and *p* < 0.05), in black for *V. v.* subsp. *sylvestris* and in gray for *V. v.* subsp. *vinifera*.

**Figure 3:**
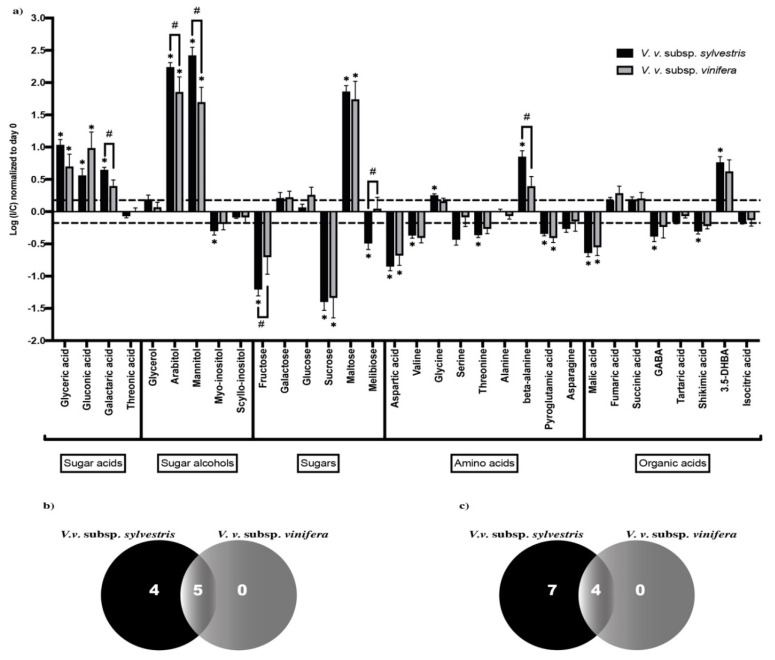
Changes in primary metabolite contents at seven DPI. (**a**) Contents are expressed as the log of the ratio between content (normalized to T0) in inoculated sample (I) and content in mock-inoculated control sample (C). Metabolite contents are represented in black for *V. v.* subsp. *sylvestris* and in gray for *V. v.* subsp. *vinifera*. * means a statistically significant difference between I and C (fold change at least ± 1.5 and *p* < 0.05). # represents a (I/C) statistically significantly different between *V. v.* subsp. *sylvestris* and *V. v.* subsp. *vinifera* (*p* < 0.05). Error bars represent standard error of the mean. (**b**) Venn diagram of metabolites increasing significantly between I and C (fold change at least ± 1.5 and *p* < 0.05) in black for *V. v.* subsp. *sylvestris* and in gray for *V. v.* subsp. *vinifera*. (**c**) Venn diagram of metabolites decreasing significantly between I and C (fold change at least ± 1.5 and *p* < 0.05), in black for *V. v.* subsp. *sylvestris* and in gray for *V. v.* subsp. *vinifera*.

To get insight into metabolite differences between *V. v.* subsp. *sylvestris* and *V. v.* subsp. *vinifera*, we analyzed mean normalized levels of each metabolite in the different *V. v.* subsp. *sylvestris* and *V. v.* subsp. *vinifera* accessions. Metabolites that are significantly impacted by the infection were identified by an increase or decrease of at least 1.5 in the fold change (FC) ratio between content in I and C samples. Significant differences between I and C were analyzed by a Wilcoxon test (*p* < 0.05) at three and seven DPI (Figure 2a and Figure 3a, respectively).

Three days after inoculation, *N. parvum* triggered enhanced levels in sugar acids (glyceric, gluconic, and galactaric acids), especially in the *V. v.* subsp. *sylvestris* genotype. Sugar alcohol contents, such as mannitol and arabitol, were strongly enhanced at three DPI, whereas sugar contents (fructose, glucose, sucrose) rather decreased, except for maltose, which increased after infection. Concerning amino acids, *N. parvum* infection triggered a decrease in aspartic acid, serine, and asparagine, and an increase in glycine, alanine, and β-alanine. Finally, for organic acids, *N. parvum* infection resulted in a decrease in malic acid but promoted the accumulation of succinic acid, γ-aminobutyric acid (GABA), and 3,5-dihydroxybenzoic acid (3.5-DHBA) (Figure 2a).

At seven days post-inoculation, primary metabolite alterations followed the same trend compared to three DPI with some modifications (Figure 3a): arabitol and mannitol contents were enhanced and higher compared to three DPI. Decrease in fructose and sucrose was also more dramatic compared to three DPI, whereas glucose content was not significantly altered and maltose raised. Concerning amino acids, we could observe a decrease in almost all of the studied amino acids, except for glycine, which was not strongly disturbed, and for β-alanine, which was significantly enhanced in *V. v.* subsp. *sylvestris*. Finally, levels of GABA, which showed an increase at three DPI, tend to decrease at seven DPI compared to mock-inoculated samples.

### 2.2. Primary Metabolic Pathways Impacted by N. parvum Infection

The overall alterations in studied primary metabolites highlighted the pathways impacted by *N. parvum* infection. Among the studied metabolites, 22 are involved in carbohydrate metabolism pathways such as the TCA cycle, glyoxylate and dicarboxylate metabolism, starch and sucrose metabolism, and galactose metabolism, etc. (Table S2). Among these 22 metabolites, we could observe that 11 increased (glyceric acid, gluconic acid, galactaric acid, glycerol, arabitol, mannitol galactose, glucose, maltose, glycine, and fumaric acid), eight decreased (myo-inositol, scyllo-inositol, fructose, sucrose, serine, malic acid, tartaric acid, and isocitric acid), and three were not significantly affected during the infection (threonic acid, melibiose, and succinic acid). Twelve studied metabolites are also involved in amino acid metabolism pathways: alanine, aspartic acid, and glutamic acid metabolism; glycine, serine, and threonine metabolism; phenylalanine metabolism; and cysteine and methionine metabolism, etc. (Table S3). Among these 12 metabolites, we could observe that four increased (glyceric acid, glycine, alanine, and fumaric acid), six decreased (aspartic acid, valine, serine, threonine, asparagine, and shikimic acid), and one did not decrease nor increase during the infection (succinic acid).

### 2.3. N. parvum Inoculation Resulted in More Intense Primary Metabolism Alterations in V. v. subsp. sylvestris Compared to V. v. subsp. vinifera

Generally, we observed the same trend in primary metabolite increase or decrease in both *V. v.* subsp. *sylvestris* and *V. v.* subsp. *vinifera*. However, we could observe a tendency for stronger primary metabolite alterations in *V. v.* subsp. *sylvestris* compared to *V. v.* subsp. *vinifera*, both three and seven days after inoculation with *N. parvum*.

Analysis of the three DPI Venn diagram (Figure 2b) revealed that among the 12 metabolites that increased significantly, five increased in both *V. v.* subsp. *sylvestris* and *V. v.* subsp. *vinifera* and seven increased only in *V. v.* subsp. *sylvestris*. Concerning the five metabolites that decreased significantly, two decreased in both *V. v.* subsp. *sylvestris* and *V. v.* subsp. *vinifera* and three decreased only in *V. v.* subsp. *sylvestris* (Figure 2c). In the Venn diagram at seven DPI (Figure 3b), we could observe that nine metabolites increased and 11 decreased in a significant way. Among the nine that significantly increased, five raised in both *V. v.* subsp. *sylvestris* and *V. v.* subsp. *vinifera* and four specifically in *V. v.* subsp. *sylvestris*. For metabolites that significantly decreased, four declined in both *V. v.* subsp. *sylvestris* and *V. v.* subsp. *vinifera* and seven only in *V. v.* subsp. *sylvestris* (Figure 3c). Together, these results may indicate a more intense response concerning primary metabolite changes in *V. v.* subsp. *sylvestris* compared to *V. v.* subsp. *vinifera*.

In addition, comparison of the I/C ratio revealed some significant differences between *V. v.* subsp. *sylvestris* and *V. v.* subsp. *vinifera* genotypes (*p* < 0.05, Figure 2a and Figure 3a). In particular, the content in arabitol, alanine, and galactaric acid tended to be higher in *V. v.* subsp. *sylvestris* compared to *V. v.* subsp. *vinifera* at three DPI. At seven DPI, the content in galactaric acid, beta-alanine, mannitol, and to a lesser extent arabitol tended to be higher in *V. v.* subsp. *sylvestris* compared to *V. v.* subsp. *vinifera*. In contrast, fructose content was lower in *V. v.* subsp. *sylvestris* compared to *V. v.* subsp. *vinifera* (Figure 3a).

### 2.4. Changes in Specialized Metabolites in Wood upon N. parvum Inoculation

**Figure 4:**
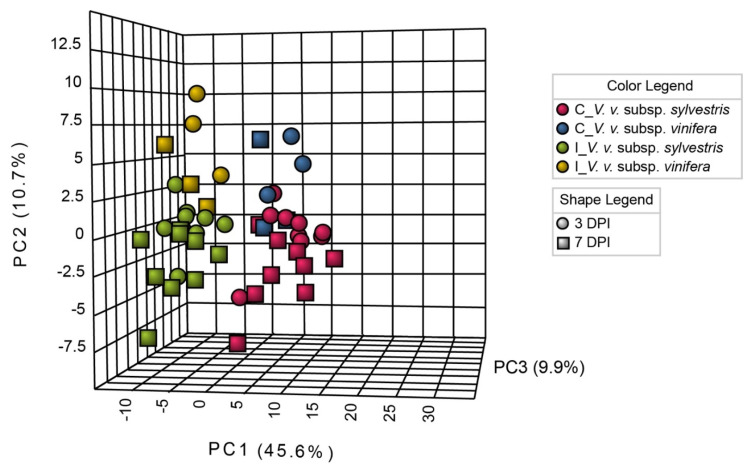
Three-dimensional principal component analysis (3D-PCA) of specialized metabolite contents. PCA was performed on mean values of the three biological replicates at three and seven days post-inoculation (DPI). The value of each biological replicate was normalized to T0 value in non-inoculated sample. Control samples (C) were inoculated with PDA, inoculated samples (I) were inoculated with *N. parvum*. Each time point is represented by a symbol (dots for three DPI and rectangles for seven DPI). The *V. v.* subsp. *sylvestris* and *V. v.* subsp. *vinifera* genotypes are represented by different colors (red for *V. v.* subsp. *sylvestris* and blue for *V. v.* subsp. *vinifera* for C samples and green for *V. v.* subsp. *sylvestris* and yellow for *V. v.* subsp. *vinifera* for I samples). The three major principal components explained 66.2% of the cumulative variance.

Specialized metabolites were analyzed by a non-targeted approach on three biological replicates conducted in 2017, 2018, and 2019: 362 metabolites were detected over the three years. Among them, 52 were found in each of the three years. In order to see the effects of infection on specialized metabolism, 3D-PCA was performed (Figure 4).

3D-PCA analysis revealed that infected and control samples were clustered in different groups, thus showing an effect of the infection. Within infected and control samples, it could be noticed that there was no clear separation between samples for three and seven DPI, suggesting a similar response at three and seven DPI concerning specialized metabolites.

**Figure 5:**
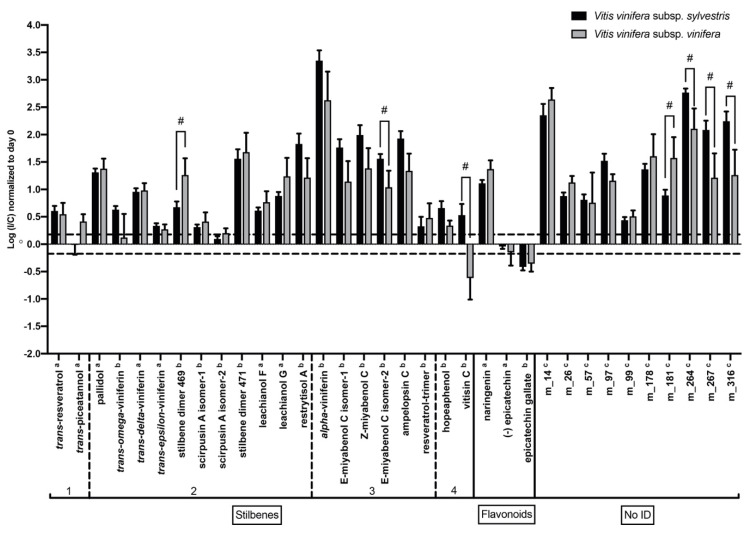
Changes in specialized metabolite contents significantly affected at three DPI. Contents are expressed as the log of the ratio between content (normalized to T0) in inoculated sample (I) and content in mock-inoculated control sample (C). Metabolite contents are represented in black for *V. v.* subsp. *sylvestris* and in gray for *V. v.* subsp. *vinifera*. # represents a (I/C) statistically significantly different for *V. v.* subsp. *sylvestris* and *V. v.* subsp. *vinifera* (*p* < 0.05). ^a^ indicates confirmed identification with standard, ^b^ indicates putative metabolites identified by MS/MS, and ^c^ indicates non-characterized metabolites. Error bars represent standard error of the mean. For stilbenes, 1-2-3 and 4 indicates monomeric, dimeric, trimeric and tetrameric forms, respectively.

**Figure 6:**
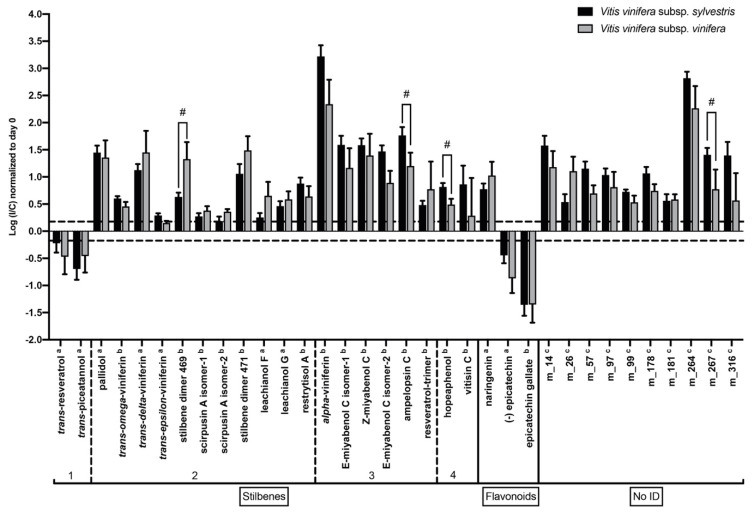
Changes in specialized metabolite contents at seven DPI. Contents are expressed as the log of the ratio between content (normalized to T0) in inoculated sample (I) and content in mock-inoculated control sample (C). Metabolite contents are represented in black for *V. v.* subsp. *sylvestris* and in gray for *V. v.* subsp. *vinifera*. # represents a (I/C) statistically significantly different for *V. v.* subsp. *sylvestris* and *V. v.* subsp. *vinifera* (*p* < 0.05). ^a^ indicates confirmed metabolites by standard, ^b^ indicates putative metabolites identified by MS/MS, and ^c^ indicates non-characterized metabolites. Error bars represent standard error of the mean. For stilbenes, 1-2-3 and 4 indicates monomeric, dimeric, trimeric and tetrameric forms, respectively.

To get insight into specialized metabolite differences between *V. v.* subsp. *sylvestris* and *V. v.* subsp. *vinifera*, we analyzed mean normalized levels of each metabolite in *V. v.* subsp. *sylvestris* and *V. v.* subsp. *vinifera* accessions. Metabolites significantly impacted by the infection were identified by a fold change ratio of at least ± 1.5 between contents in infected (I) and control (C) samples normalized to T0 and by a Wilcoxon test (*p* < 0.05) at three and seven DPI. Among the 52 metabolites detected in each of the three years (Figures S1 and S2), 34 were significantly altered (increase or decrease) at three and seven DPI for *V. v.* subsp. *sylvestris* and/or *V. v.* subsp. *vinifera* (Figure 5 and Figure 6). Of these 34 metabolites, seven were identified at level 1 on the Schymanski scale and 27 at level 5 [24]. Among these 27 metabolites, ten did not match with our databases and 17 had a putative identification (Grapecyc, KEGG, homemade database). In order to confirm this identification, MS2 analyses were performed. After analysis of the ten, 25, and 40 eV fragmentation profiles, we were able to further identify 15 metabolites using MS-Finder [25,26], Global Natural Products Social Molecular Networking (GNPS) [27], and literature [28,29,30,31,32,33] (Table S4). The compounds significantly altered by *N. parvum* infection were assigned thanks to high-resolution mass analysis, MS/MS fragmentation patterns, and UV-vis absorption spectra. The identification of stilbene tetramers m_293 and m_300 has been done according to Reference [29]. Similarly, MS/MS fragmentation patterns [30] and UV data [31,32,33] allowed to identify epicatechin-3-*O*-gallate m_111, stilbene dimers (m_125, m_152, m_153 and m_158), and trimers (m_235; m_236, m_239, m_241 and m_242).

Among the 34 metabolites significantly impacted by *N. parvum* infection, we found stilbenes, which are known phytoalexins in grapevine (2 monomers, 11 dimers, six trimers, and two tetramers), flavonoids (three compounds), and ten compounds that we could not associate to a known family due to a lack of characterization (Figure 5 and Figure 6). At three DPI, we could measure a significant increase in different stilbenes in *V. v.* subsp. *sylvestris* and *V. v.* subsp. *vinifera*, especially *trans*-resveratrol, pallidol, *trans*-δ-viniferin, α-viniferin, several resveratrol trimers, and tetramers including hopeaphenol. On the other hand, contents in flavonoids such as epicatechin and epicatechin-3-*O*-gallate rather decreased, whereas content in naringenin significantly increased (Figure 5).

At seven DPI, we could observe the same trend as for three DPI, except for *trans-*resveratrol and *trans*-piceatannol, which decreased at a later time point (Figure 6).

### 2.5. Response to N. parvum in V. v. subsp. sylvestris Is Characterized by Higher Induction of Resveratrol Oligomer Levels after Wood Inoculation

Response in specialized metabolite alterations appears to be rather similar in *V. v.* subsp. *sylvestris* and *V. v.* subsp. *vinifera* with changes in compounds notably belonging to stilbene and flavonoid families. However, significant differences in the I/C ratio for different specialized metabolites were observed between *V. v.* subsp. *sylvestris* and *V. v.* subsp. *vinifera* (*p* < 0.05). Levels of 2 resveratrol oligomers are generally more strongly induced at three DPI in the wood of *V. v.* subsp. *sylvestris* (Figure 5): the trimer E-miyabenol C isomer-2 (m_241) had a higher I/C ratio in *V. v.* subsp. *sylvestris*, and contents in the tetramer vitisin C were induced by infection in *V. v.* subsp. *sylvestris,* whereas they decreased in *V. v.* subsp. *vinifera*. In contrast, I/C ratio for the stilbene dimer 469 (m_150) was significantly higher in *V. v.* subsp. *vinifera*. Four other unknown metabolites showed a differential accumulation in *V. v.* subsp. *sylvestris* and *V. v.* subsp. *vinifera* at three days post-inoculation compared to control (Figure 5): one of them (m_181) was more induced in *V. v.* subsp. *vinifera*, and three of them (m_264, m_267 and m_316) had a higher I/C ratio in *V. v.* subsp. *sylvestris*.

Seven days post-inoculation, a lower number of metabolites showed significant different induction in the two genotypes compared to three DPI. Among the seven significantly different metabolites at three DPI, only two remained different at seven DPI: stilbene dimer 469 (m_150) was more induced in *V. v.* subsp. *vinifera* and m 267 had a higher I/C ratio in *V. v.* subsp. *sylvestris*. Interestingly, two new resveratrol oligomers showed different induction at seven DPI in the two genotypes: the trimer ampelopsin C (m_242) and the tetramer hopeaphenol (m_293) were more strongly induced in *V. v.* subsp. *sylvestris* compared to *V. v.* subsp. *vinifera*. These results show an early different specialized metabolite induction between the two genotypes.

## 3. Discussion

*V. v.* subsp. *sylvestris,* which is considered to be the progenitor of cultivated grapevines, has important tolerance potential for abiotic stress [34] and pathogen resistance [23,35]. However, mechanisms underlying stress tolerance in this subspecies are still not explored in detail. Interaction between grapevine and trunk disease-associated fungi is also poorly understood, especially in the wood. Previous metabolomic studies of grapevine infected by trunk disease fungi were performed on leaves [15,18]. However, to gain a better understanding of mechanisms underlying trunk disease progression, there is a need to study metabolic perturbations in the wood. Our study shows that both primary and specialized metabolites undergo important changes in the wood following infection with *N. parvum*. We are nevertheless aware that in contrast to studies on leaves, where perturbations rather reflect plant metabolism, metabolomic studies on wood reflect metabolites from both plant and fungal origin.

Concerning primary metabolites, *N. parvum* infection triggered a strong decrease in sugars such as fructose, glucose, and sucrose and an increase in maltose. Increase in maltose was also reported in the case of Rosaceae infection with the biotrophic pathogen *Gymnosporangium asiaticum* [36]. It is possible that maltose arises from starch degradation during infection since a depletion in starch reserves has been observed in wood infected by GTD fungi [6]. On another hand, decline of sugars is a hallmark of pathogen attack since the fungus acts as an additional sink within the plant tissue and diverts sugars, especially hexoses, to its own profit [37]. A decrease in carbohydrates has been described in the case of leaves from *V. vinifera* cv. Alvarino affected by esca [15] and could result from rerouting of sugars toward specialized metabolite synthesis. In contrast, Moret et al. [18] found a higher level in carbohydrates such as glucose and fructose in leaves of Chardonnay clone 95 plants showing esca symptoms. In our study, decrease of sugars we observed in the wood could thus also be explained by remobilization toward the leaves to better sustain the infection. In a recent study, Cardot et al. [38] showed coordinated upregulation of the hexose transporter *VvHT5* and cell wall invertase in leaves of the tolerant cultivar Merlot elicited by *Eutypa lata*, but not in the highly susceptible cultivar Ugni Blanc. Regulation of sugar availability thus seems crucial for plant tolerance to trunk diseases.

Concerning amino acids, *N. parvum* infection triggered a decrease in aspartic acid, serine, and asparagine, and a strong increase in alanine and β-alanine at three DPI. Alanine and β-alanine accumulation were higher in *V. v.* subsp. *sylvestris* compared to *V. v.* subsp. *vinifera* at three and seven DPI, respectively. Accumulation of alanine has been previously reported in Chardonnay leaves just before esca apoplexy [14]. Several amino acids (asparagine, isoleucine, leucine, methionine, phenylalanine, proline, tyrosine, and valine) were also found to increase in the xylem sap of Chardonnay undergoing both water stress and esca disease [39]. Alterations in amino acid contents thus seem to be a common feature of trunk diseases. The exact function of amino acids in plant response to pathogens is not well established. β-Alanine accumulation has been reported in several plants submitted to biotic and abiotic stresses: in opium poppy cells, β-alanine was only accumulated in elicitor-treated cultures [40] and also accumulated in MeJA-treated *Medicago truncatula* cells [41]. It has been proposed that β-alanine could play diverse roles: as nitrogen reserve, in plant defense signaling, protein and membrane stabilization, and in the maintenance of osmotic pressure [15,42,43].

GABA, a non-protein amino acid, showed a significant increase in *V. v.* subsp. *sylvestris* and *V. v.* subsp. *vinifera* wood at three DPI. GABA could be involved in various biological and physiological responses to stress conditions [44]. A number of pathogenic fungi are also able to synthesize GABA. In the wheat fungal pathogen *Stagonospora nodorum*, GABA is implicated in response to environmental stimuli and asexual development and differentiation [45]. In plants, GABA, together with the secondary messenger Ca^2+^, could be involved in defense response signaling [44].

Both at three and seven DPI, a strong increase in sugar alcohols (mannitol and arabitol) was observed. Both sugar alcohols could be of fungal origin since mannitol has been described as a common carbohydrate stored in fungi. Indeed, it has been demonstrated that the necrotrophic pathogens *Botrytis cinerea* and *Sclerotinia sclerotiorum* convert plant hexoses, especially glucose, to mannitol, which is the main energy store in *B. cinerea* [46,47]. Moreover, mannitol, as a reactive oxygen species (ROS) quencher, may be used by pathogenic fungi to sustain ROS produced by the plant as a defense mechanism [47]. On another hand, accumulation of polyols such as mannitol was observed in a variety of plants submitted to stresses, especially water stress, and could protect cells from deleterious osmotic and metabolic imbalances [48]. Conde et al. [49] demonstrated that mannitol accumulation is crucial for salt and osmotic stress tolerance in *Olea europea*. In addition, mannitol accumulation induced by overexpression of an *Escherichia coli* mannitol-1-phosphate dehydrogenase resulted in increased tolerance to water stress and salinity in several plants [50]. A trend for higher levels in both arabitol and mannitol was observed in *V. v.* subsp. *sylvestris* compared to *V. v.* subsp. *vinifera* after infection. Since GTD pathogens colonize the vascular system, they cause vessel occlusion and a decline in hydraulic conductivity, as it is observed in the case of water stress. We can thus hypothesize that higher polyol levels observed in *V. v.* subsp. *sylvestris* may help the plant to sustain the stress caused by *N. parvum*.

Overall, primary metabolite signature indicates that a higher number of metabolites exhibit significant changes after *N. parvum* inoculation in *V. v.* subsp. *sylvestris* compared to *V. v.* subsp. *vinifera*, especially at 3 DPI. A faster and stronger metabolic reprogramming could thus allow the *V. v.* subsp. *sylvestris* genotype to better sustain a pathogen attack. 

Analyses of specialized metabolites showed that major changes concerned compounds belonging to stilbene and flavonoid classes, which are rather specific from plants. A correlation between the accumulation of flavonoids, hydroxycinnamoyl tartaric acids, and esca-disease expression was also reported by Lima et al. [17]. Concerning flavonoids, content in naringenin was stimulated by infection, whereas contents in catechin and particularly epicatechin and epicatechin-3-*O*-gallate decreased. Catechin and epicatechin, which are the precursors of condensed tannins, could be used for the synthesis of these antimicrobial compounds.

Stilbenes, which are well-known phytoalexins in grapevine, showed major alterations in the wood after inoculation with *N. parvum*. A number of studies involved stilbenes in both inducible and constitutive plant defenses to bioagressors [51]. There are also several reports indicating that stilbenes could participate in the grapevine response to trunk diseases. Accumulation of phytoalexins (resveratrol and derivatives such as *ε*-viniferin) was previously reported in different tissues or organs of grapevine affected by esca [52]. In addition, *V. v.* subsp. *vinifera* cells treated with secreted proteins from *N. parvum* accumulated high levels of δ-viniferin [53]. More complex stilbenoids such as ampelopsins A, B, and H, leachianols F and G, hopeaphenol, isohopeaphenol, and pallidol were found higher in discolored wood of esca-diseased vines [52,54]. Induction of genes involved in resveratrol biosynthesis pathway in esca-diseased leaves prior to symptom appearance was also observed [14]. In addition, comparison between cultivars with contrasted sensitivity to esca also revealed that less susceptible cultivars showed an earlier and stronger induction of the *PAL* and *STS* genes, and a higher accumulation of stilbene compounds compared to a highly susceptible one [55].

In this study, three days after inoculation, the content in resveratrol and several resveratrol oligomers (resveratrol dimers, trimers, and tetramers) increased in the wood of both *V. v.* subsp. *vinifera* and *V. v.* subsp. *sylvestris* genotypes compared to non-infected wood. However, seven days after inoculation, the I/C ratio of resveratrol and piceatannol decreased, whereas the I/C ratio for resveratrol oligomers remained high. This suggests that these two stilbene monomers are consumed for the oligomer synthesis at later time points after infection. It is interesting to point out that content in 3,5-DHBA, which could be a biodegradation product of resveratrol, increased at both three and seven DPI (Figure 2 and Figure 3). It is thus possible that *N. parvum* can degrade resveratrol as it has been observed for the fungal endophyte *Phomopsis liquidambari* [56].

Importantly, the I/C ratio of some stilbene oligomers was shown significantly different between *V. v.* subsp. *sylvestris* and *V. v.* subsp. *vinifera*. Levels in resveratrol dimers and trimers were significantly induced by *N. parvum* infection in the wood of both *V. v.* subsp. *sylvestris* and *V. v.* subsp. *vinifera* genotypes. However, E-miyabenol C isomer-2 and ampelopsin C were more induced in the wood of *V. v.* subsp. *sylvestris* compared to *V. v.* subsp. *vinifera* at three and seven DPI, respectively. On another hand, the I/C ratio of the stilbene dimer 469 was higher in *V. v.* subsp. *vinifera*. Interestingly, the content in the tetramer vitisin C was upregulated at three DPI in *V. v.* subsp. *sylvestris* genotype but decreased in *V. v.* subsp. *vinifera*. Several resveratrol oligomers were previously described in the wood of *V. vinifera* cv. Sangiovese affected by esca proper [52]. Dimers, trimers, and tetramers of resveratrol were present both in the wood of asymptomatic and symptomatic grapevines [52]. Interestingly, comparison of the resveratrol oligomer profile between asymptomatic and brown symptomatic wood showed higher contents in several resveratrol oligomers (such as the tetramer hopeaphenol and the dimers ampelopsin A and B) in symptomatic brown wood. In another study, Lambert et al. [57] reported an increase in resveratrol oligomers (ε-viniferin, miyabenol C, hopeaphenol, and isohopeaphenol) in foliar cuttings inoculated with *N. parvum*, whereas the monomers resveratrol, piceid, and piceatannol did not vary significantly. It is known that dimers (viniferins) and oligomers of resveratrol have a potent antifungal activity, greater than the resveratrol monomer [51]. In the case of trunk disease pathogens, Stempien et al. [58] have shown that resveratrol has low fungistatic activity on fungi associated with Botryosphaeria dieback (*N. parvum* and *Diplodia seriata*), which are able to rapidly metabolize this compound. In contrast, δ-viniferin exhibited a significantly higher fungistatic activity especially toward *N. parvum* Bourgogne [58]. Botryosphaeriaceae fungi thus appear to have lower metabolization activity of resveratrol oligomers, although they are able to grow in the wood containing these compounds. Interestingly, Lambert et al. [57] showed that hydroxystilbenoids, especially oligomers such as miyabenol C, isohopeaphenol, and vitisin A and B, significantly reduced the growth of GTD-associated fungi, especially Botryospaheriaceae. Even if these compounds are fungistatic and not fungicidal, it is thus possible that higher inducibility of several resveratrol oligomers is part of the better tolerance of the *V. v.* subsp. *sylvestris* genotypes to GTD infection. Antimicrobial stilbene signature thus seems to be a hallmark of pathogen tolerance evidenced in this genotype. Indeed, *V. v.* subsp. *sylvestris* genotypes showing quick and strong accumulation of resveratrol and viniferins in the leaves following UV stress had a lower sensitivity to *Plasmopara viticola*, the downy mildew agent [35].

In conclusion, metabolic response of *V. v.* subsp. *sylvestris* to *N. parvum* infection is characterized by more rapid and intense alteration in primary metabolites compared to *V. v.* subsp. *vinifera* and by a higher induction of several resveratrol oligomer contents. Both of these responses could participate in the better tolerance of *V. v.* subsp. *sylvestris* to trunk diseases. Several other unknown metabolites are also differently accumulated in the two genotypes and future work should focus on their elucidation in order to better understand the wood response to GTDs.

## 4. Materials and Methods 

### 4.1. Plant Material

Detached internodes of 1-year old dormant lignified canes were used for inoculation and were collected from three *V. v.* subsp. *vinifera* commercial cultivars (Chardonnay, Müller-Thurgau, and Gewürztraminer) and nine *V. v.* subsp. *sylvestris* accessions (Ke 2, Ke 34, Ke 53, Ke 83, Ke 84, Ke 95, Ke 106, Ke 119, and Hö 29). The Ke accessions originate from a large population of *V. v.* subsp. *sylvestris* on the Ketsch peninsula in Germany and the Hördt accession was from an isolated plant in Hördt alluvial forest (Upper Rhine valley, Rheinland-Pfalz). These accessions belong to an ex situ conservation program and are grown in the Botanical Garden of the Karlsruhe Institute of Technology. This collection constitutes part of a complete genetic copy of the *V. v.* subsp. *sylvestris* still existing in Germany, and accessions have been characterized genetically by microsatellite markers [59]. Three biological replicates were analyzed over three years (2017, 2018, and 2019). For each year, four internodes of each accession were inoculated for each time point and treatment.

### 4.2. Fungal Material and Wood Inoculation

*N. parvum* Bt-67 was obtained from a single spore collection of the Instituto Superior de Agronomia (Universidade de Lisboa, Portugal). *N. parvum* was grown on potato dextrose agar (PDA) at 27 °C in the dark and was subcultured every ten days. Internodes from dormant canes were used for wood inoculation experiments according to the protocol adapted from Guan et al. [23]. Woody internodes (Ø 7 mm) were drilled at the center with a power drill (PSB 500R, Bosch, Saint-Ouen, France) (Ø 5 mm and 3 mm deep). For each biological replicate, four internodes of each genotype were inoculated with a 5 mm diameter plug collected from a 14-day-old fungal culture on potato dextrose agar. The inoculation point was subsequently sealed with Parafilm. Mock-inoculated internodes were inoculated with PDA plugs. Non-inoculated intact internodes (T0 samples) were collected, freezed in liquid nitrogen, and freeze-dried after debarking and cutting in a lyophilizer (Alpha 1–2 LD freeze dryer, Christ, Osterode am Harz, Germany). After inoculation, the detached internodes were incubated in the dark at 28 °C in a saturated humidity chamber. Samples were collected after 3–7 days of incubation: the stems were debarked and wood sections of 1.5 cm long were made from 0.5 cm of the drill point; on both sides. Wood sections were then cut in approximately 2 mm thick sections, freeze-dried in a lyophilizer (Alpha 1–2 LD freeze dryer, Christ, Osterode am Harz, Germany), and then ground for 2 min at speed 10 using a planetary mill (MiniMill, Philips, Eindhoven, The Netherlands) with bowl and four beads (1 cm diameter) made from zirconium oxide.

### 4.3. Metabolite Extraction

Approximately 10 mg of ground woody tissue was precisely weighted and aliquoted in 2 mL polypropylene microtubes for successive extractions of primary and secondary metabolites. For extraction of primary metabolites, woody powder was first extracted with 600 µL of 50 mM potassium phosphate buffer pH 6 containing phenoxyacetic acid at 10 mg/L as an internal standard, in an ultrasound bath during 30 min (power 9, 20 °C as starting temperature). Samples were centrifuged (20,000 g, 20 min, 20 °C). Then, the supernatant was deposited on a filtration plate (96-well plates Acroprep 1 mL, 0.45 µm GHP membrane, Pall Life Science, Portsmouth, United Kingdom) and filtered with the help of a vacuum manifold (Pall Corporation, Portsmouth, United Kingdom). The filtrate was collected on a collector plate (96-well PP plate, 1.2 mL, VWR 732-2890/391-0077, Fontenay-sous-Bois, France). Two further similar extractions were performed on the pellet, a second with the same extraction buffer and a third with LC-MS grade water (Fisher Scientific, Illkirch, France), to rinse the sample as much as possible. The steps of sonication, centrifugation, and filtration remained the same for all extractions. Extraction 1 and 2 were pooled together, agitated on a Titramax 1000 orbital shaker (Heidolph Instruments GmbH & CO., Schwabach, Germany) at 1350 rpm for 1 min, and used for GC-MS analysis (Section 4.4). The remaining pellet after extractions was freeze-dried and used for specialized metabolite extraction.

For extraction of specialized metabolites, we performed a one-step extraction. The remaining pellet was extracted in an ultrasound bath (power 9 and 20 °C as starting temperature) with 500 µL of LC-MS grade methanol (Fisher Scientific, Illkirch, France) containing 5-methylsalicylic acid at 5 mg/L as an internal standard. The steps of sonication, centrifugation, and filtration remained the same as those described above. The obtained filtrates were analyzed by LC-MS Section 4.5).

### 4.4. GC-MS Analysis

Thirty microliters of each extract were freeze-dried in an amber HPLC vial. Prior to GC-MS analysis, a derivatization reaction was carried out on “non-diluted” and “diluted” extracts according to the method inspired by Noctor et al. [60]. Twenty microliters of methoxyamine hydrochloride solution at 30 mg/mL in anhydrous pyridine were subsequently added in each vial, which was placed at 37 °C and 600 rpm for 120 min. Eighty microliters of n-methyl-n-(trimethylsilyl)trifluoroacetamide (MSTFA) were added and samples were incubated for 30 additional minutes at 37 °C and 600 rpm. Finally, samples were stored at room temperature for 60 min before GC-MS injection. The injection volume of samples and calibrants was 1 µL.

Quantitative GC-MS analysis of wood extracts was performed on GC-2010 gas chromatography coupled with GC-QP2010 mass detector (Shimadzu corporation, Tokyo, Japan) operating at 70 eV ionization source. In order to separate the compounds, an SGE analytical Science BPX5 column (25 m × 0.25 µm, Ø 15 mm) was used. The MS was adjusted using perfluorotributylamine (PFTBA). The helium flow rate was 0.97 mL/min. The initial temperature of the column was 110 °C, increased to 155 °C at a rate of 10.5 °C/min, then increased to a final temperature of 350 °C at a rate of 11.5 °C/min. The oven was maintained at this temperature for 6 min. The injector was set at 310 °C, the transfer line at 330 °C, and the ion source at 200 °C. The total analysis time was 31.24 min. Two selected ion monitoring (SIM) methods described in supplementary data were used for the diluted and non-diluted samples (Tables S5 and S6). Data were acquired with Shimadzu Real-Time Analysis version 4.5 (Shimadzu corporation, Tokyo, Japan) system software. The absolute quantification of the primary metabolites was performed thanks to an external calibration. The external calibration was performed by mixing 32 commercially available compounds at 1 mM concentration in water. Six calibration standard solutions (25, 10, 5, 2.5, 1.25, and 0.5 µM) were made by serial dilution from this stock solution. Calibrants were analyzed in triplicate according to the same protocol as the extracts.

### 4.5. HPLC-DAD-MS Analysis

The analytical system used was High-Performance Liquid Chromatography Agilent 1100 series equipped with a diode array detector (DAD) and coupled to Agilent 6510 accurate-mass Quadrupole-Time of Flight (Q-TOF) mass spectrometer with electrospray ionization (ESI) interface in negative ionization mode (Agilent Technologies, Santa Clara, CA, USA). The mobile phase solvents were composed of 0.1% formic acid in LC-MS grade water (solvent A) and 0.1% formic acid in LC-MS grade methanol (solvent B) to separate secondary metabolites on a Zorbax SB-C18 column (3.1 × 150 mm, Ø 3.5 µm), equipped with a Zorbax Eclipse plus C18 pre-column (2.1 × 12.5 mm, Ø 5 µm) (Agilent Technologies, Santa Clara, CA, USA). The gradient solvent system was composed of ratios of solvent A and B: 95:5 (0–3 min), 95:5 to 0:100 (3–23 min), 0:100 (23–33 min), and 95:5 (33–40 min). The flow rate was 0.35 mL/min. The injection volume of samples was 2 µL. The drying gas flow and the nebulizer pressure were set at 13.0 L/min at 325 °C and 35 psi, respectively. Other MS conditions included fragmentor: 150 V, capillary voltage: −3500 V and collision energy: 20 V. Negative mass calibration was performed with a mix of standard compounds (G1969-85000, Agilent Technologies, Santa Clara, CA, USA). Data were acquired with Agilent MassHunter version B.02.00 system software. MS/MS experiments were carried out at 10, 25, and 40 eV with the analytical method previously described and nitrogen as collision gas.

### 4.6. Metabolomic Data Processing and Statistical Analysis 

In order to highlight metabolomic responses of the *V. v.* subsp. *vinifera* and *V. v.* subsp. *sylvestris* groups, the different accessions and cultivars belonging to each specific group were pooled together for analysis. The GC-MS raw data processing was performed with Shimadzu post-run analysis from the Shimadzu lab solution with the following parameters for peak integration: Savitzky-Golay smoothing method [61] (# of peaks:5, width:9). The result table was imported into Microsoft Excel prior to calculate the exact concentration according to the exact weighted mass of the sample and the standard calibration curve. Data pretreatment, multivariate, and univariate statistical analyses were performed with the MetaboAnalyst 4.0 online platform (https://www.metaboanalyst.ca, [62]). As data pretreatment, the metabolite contents were submitted to different steps of normalization: firstly, they were normalized to the internal standard, secondly to the content of intact canes (T0), and finally by the pareto scale method [63] (each metabolite was mean-centered and divided by the square of the standard deviation).

To point out the impact of the fungal infection on metabolite contents, a 1.5-fold change ratio analysis was applied, and a Wilcoxon test (*p* < 0.05) was performed to identify specific metabolites significantly impacted by the treatment (Infected or Control) at a specific time point. A Wilcoxon test analysis was performed on the I/C ratio to identify specific metabolites significantly impacted by the subspecies (*V. v.* subsp. *sylvestris*/*V. v.* subsp. *vinifera*) at a specific time point. A false discovery rate approach was performed as a multiple testing correction. A three-dimensional principal component analysis (3D-PCA) was performed on centered-reduced data. The heatmaps represent the effect of the fungal infection between normalized I and C samples (I/C ratio). Histograms are drawn using Prism 8 (version 8.2.1). Venn diagram of impacted metabolites (*p* < 0.05 and FC ± 1.5) are drawn using jvenn online platform [64]. Error bars on histograms represent the standard error of the mean [65].

The HPLC-MS raw data pre-processing was performed with Agilent Profinder software version B.07.00 and consisted of feature extraction combined with peak alignment and integration. The preprocessed datasets, corresponding to 362 features in negative mode over the three years, were imported in Microsoft Excel and converted to .txt files. Data pretreatment consists of a baseline correction to the median, normalization by the internal standard, and a pareto scale method (each metabolite was mean-centered and divided by the square of the standard deviation). Prior to the statistical analysis, datasets were filtered based on the 80% modified rules [66] to remove metabolites with low reproducibility. The same statistical analysis as for primary metabolites was carried out for the secondary metabolites. Metabolite characterization was performed on all detected metabolites, first with confirmed standard. A MS/MS identification was carried out on significantly affected compounds, and the MS/MS spectra were analyzed with MS-Finder [25,26] and GNPS [27].

## Supplementary Materials

The following are available online at https://www.mdpi.com/2218-1989/10/6/232/s1, Figure S1: Changes in specialized metabolite contents at three DPI, Figure S2: Changes in specialized metabolite contents at three DPI, Table S1: List of primary metabolites studied by GC/MS, Table S2: List of primary metabolites involved in cabohydrate metabolism, Table S3: List of primary metabolites involved in amino acid metabolism, Table S4: List of the 34 secondary metabolites analyzed by MS/MS and significantly impacted by *N. parvum* infection, Table S5: SIM method used for 1/20th diluted samples, Table S6: SIM method used for non-diluted samples.
