# Supplementary material for: Wood Metabolomic Responses of Wild and Cultivated Grapevine to Infection with Neofusicoccum parvum, a Trunk Disease Pathogen
Source: Metabolites. 2020 Jun 4;10(6):232. doi: 10.3390/metabo10060232 (PMC7344444; doi:10.3390/metabo10060232)
Supplement: Supplementary file 1 [file metabolites-10-00232-s001.pdf]

Table S1: List of primary metabolites studied by GC/MS. "Target ion" corresponds to the ion used for compound quantification and "ion fragments" are used to check the compound identity.

Name	Formula	Family	Molecular weigth (g/mol)	Retention time (min)	Target ion	Ion fragment
L-alanine	C3H7NO2	Amino acids	89.0932	3.7	116	100-190
L-valine	C5H11NO2	Amino acids	117.1463	5.7	144	218-79
L-glycine	C2H5NO2	Amino acids	75.0666	7.2	174	100-248
L-serine	C3H7NO3	Amino acids	105.0926	8.0	204	100-218
L-threonine	C4H9NO3	Amino acids	119.1192	8.3	218	117-101
β-alanine	C3H7NO2	Amino acids	89.0932	9.0	174	248-290
L-Aspartic acid	C4H7NO4	Amino acids	133.1027	10.2	232	100-218
Pyroglutamic acid	C5H7NO3	Amino acids	129.1140	10.5	156	230-258
L-Asparagine	C4H8N2O3	Amino acids	132.1179	12.0	116	231-188
Tartaric acid	C4H6O6	Organic acids	150.0868	11.5	292	189-219
Malic acid	C4H6O5	Organic acids	134.0874	9.9	233	133-101
Isocitric acid	C6H8O7	Organic acids	192.0270	13.4	273	375-319
Succinic acid	C4H6O4	Organic acids	118.0880	7.5	247	129-172

Fumaric acid	C4H4O4	Organic acids	116.0722	8.0	245	133-115
Gamma aminobutyric acid (GABA)	C4H9NO2	Organic acids	103.1198	10.4	174	304-100
Shikimic acid	C7H10O5	Organic acids	174.1513	13.3	204	133-255
3.5-dihydroxybenzoic acid (3.5-DHBA)	C7H6O4	Organic acids	154.1201	13.5	370	355-311
Glyceric acid	C3H6O4	Sugar acids	106.0773	7.5	189	292-103
Threonic acid	C4H8O5	Sugar acids	136.1033	10.6	292	220-205
Gluconic acid	C6H12O7	Sugar acids	196.1553	14.9	292	103-217
Mucic acid	C6H10O8	Sugar acids	210.1388	15.3	333	292-305
Glycerol	C3H8O3	Sugar alcohols	92.0473	6.6	103	117-205
Arabitol	C5H12O5	Sugar alcohols	152.1458	12.3	217	103-205
Mannitol	C6H14O6	Sugar alcohols	182.0790	14.2	319	103-205
Myo-Inositol	C6H12O6	Sugar alcohols	180.0633	15.7	305	318-191
Scyllo Inositol	C6H12O6	Sugar alcohols	180.1559	15.1	318	217-190
Fructose	C6H12O6	Sugars	180.0633	13.8	307	217-133
Galactose	C6H12O6	Sugars	180.0633	13.9	205	117-105
Glucose	C6H12O6	Sugars	180.0633	14.0	319	160-103
Sucrose	C12H22O11	Sugars	342.1162	19.6	361	437-129

Maltose	C12H22O11	Sugars	342.1162	20.2	361	204-217
Melibiose	C12H22O11	Sugars	342.2965	21.0	361	204-217

Table S2: List of primary metabolites involved in cabohydrate metabolism. Metabolite involved in specific pathways are indicated by a green box.

**Metabolite name**	**Glycolysis / Gluconeogenesis**	**Citrate cycle**	**Pentose phosphate pathway**	**Pentose and glucuronate interconversions**	**Fructose and mannose metabolism**	**Galactose metabolism**	**Ascorbate and aldarate metabolism**	**Starch and sucrose metabolism**	**Pyruvate metabolism**	**Glyoxylate and dicarboxylate metabolism**	**Inositol phosphate metabolism**
**Glyceric acid**			v							v	
**Gluconic acid**			v								
**Galactaric acid**							v				
**Threonic acid**							v				
**Glycerol**				v		v					
**Arabitol**				v							
**Mannitol**					v						
**Myo-inositol**						v	v				v
**Scyllo-inositol**											v
**Fructose**					v	v		v			

**Galactose**						v					
**Glucose**	v		v			v		v			
**Sucrose**						v		v			
**Maltose**								v			
**Melibiose**						v					
**Aspartic acid**											
**Valine**											
**Glycine**										v	
**Serine**										v	
**Threonine**											
**Alanine**											
**ß-alanine**											
**Pyroglutamic acid**											
**Asparagine**											
**Malic acid**		v							v	v	
**Fumaric acid**		v									

Succinic acid	v	v
GABA		
Tartaric acid		v
Shikimic acid		
3.5-DHBA		
Isocitric acid	v	v

Table S3: List of primary metabolites involved in amino acid metabolism. Metabolite involved in specific pathways are indicated by a green box.

Metabolite	Alanine. aspartate and glutamate metabolism	Glycine. serine and threonine metabolism	Cysteine and methionine metabolism	Valine. leucine and isoleucine degradation	Valine. leucine and isoleucine biosynthesis	Lysine degradation	Arginine biosynthesis	Arginine and proline metabolism	Tyrosine metabolism	Phenylalanine metabolism	Phenylalanine. tyrosine and tryptophan biosynthesis
Glyceric acid		v									
Gluconic acid											
Galactaric acid											
Threonic acid											
Glycerol											
Arabitol											
Mannitol											
Myo-inositol											
Scyllo-inositol											
Fructose											
Galactose											

**Glucose**											
**Sucrose**											
**Maltose**											
**Melibiose**											
**Aspartic acid**	v	v	v				v				
**Valine**				v	v						
**Glycine**		v									
**Serine**		v	v								
**Threonine**		v			v						
**Alanine**	v		v								
**ß-alanine**											
**Pyroglutamic acid**											
**Asparagine**	v										
**Malic acid**											
**Fumaric acid**							v		v	v	
**Succinic acid**	v					v			v	v	
**GABA**	v							v			
**Tartaric acid**											
**Shikimic acid**											v
**3.5-DHBA**											
**Isocitric acid**											

Table S4: List of the 34 secondary metabolites analyzed by MS/MS and significantly impacted by *N. parvum* infection. [a] indicates metabolites with confirmed identification by standard. [b] indicates putative metabolites identified by MS/MS and UV data and [c] indicates non-characterized metabolites.

Feature name	RT (min)	Molecular formula	Monoisotopic mass	Calculated $[M - H]^-$ m/z	Measured $[M - H]^-$ m/z	│Δppm│	Base Peak	Assigned compound	lmax (nm)	MS/MS fragments [1]
m_14	21.62	NA	NA	NA	211.07690		$[M - H]^-$	m_14 [c]	206. 234. 314	169.0759; 167.0924 @ 10 eV
m_15	18.18	C14H12O3	228.07865	227.07137	227.07138	0.05	$[M - H]^-$	trans-resveratrol [a]	220. 306	164.9418; 119.0411; 109.4032
m_20	16.86	C14H12O4	244.07356	243.06628	243.06656	1.14	$[M - H]^-$	trans-piceatannol [a]	222. 322	201.0527; 199.0723; 175.0734; 173.0581; 172.0501; 159.0426; 135.0440; 41.0041
m_24	20.43	C15H12O5	272.06848	271.0612	271.06187	2.48	$[M - H]^-$	naringenin [a]	216. 290	177.0191; 151.0040; 119.0500; 107.0138
m_26	18.07	NA	NA	NA	287.06322		$[M - H]^-$	m_26 [c]	218. 306	124.2962; 107.0563; 93.0416; 89.0316
m_30	14.87	C15H14O6	290.07904	289.07176	289.07243	2.30	$[M - H]^-$	(-)-Epicatechin [a]	204. 226. 278	245.0793; 227.0679; 221.0792; 205.0481; 203.0688; 202.0605; 188.0456; 187.0377; 179.0323; 175.0540; 165.0176; 164.0092; 162.0300; 161.0590; 159.0430; 151.0384; 150.0306; 149.0228; 146.0352; 145.0271; 139.0394; 138.0301; 137.0225; 135.0440; 125.0234; 123.0438; 122.0357; 121.0282; 109.0284; 97.0297; 95.0498; 93.0349; 83.0137; 81.0344; 57.0356
m_57	19.98	NA	NA	NA	315.05117		$[M - H]^-$	m_57 [c]	202. 220. 328	217.12102; 272.89789
m_97	16.47	NA	NA	NA	425.08695		$[M - H]^-$	m_97 [c]	202. 228. 284	363.0830; 275.0682; 271.0606 @ 10 eV
m_99	20.02	NA	NA	NA	427.15158		$[M - H]^-$	m_99 [c]	304. 380	393.4279; 337.0934; 307.1060; 289.0881; 265.0872
m_111	15.84	C22H18O10	442.09000	441.08272	441.08231	0.94	$[M - H]^-$	epicatechin-3-O- gallate [b]	202. 224. 282	289.0750; 271.0664; 169.0265 @ 10 eV
m_123	16.63	C28H22O6	454.14164	453.13436	453.13436	4.47	$[M - H]^-$	pallidol	202. 286	359.0923; 265.0582; 264.4424; 93.0423

m_125	19.9	C28H22O6	454.14164	453.13436	453.13509	1.60	[M - H]-	trans-w-viniferin [b]	206. 226. 320	435.1209; 409.1414; 383.1386; 369.1137; 359.0962; 347.0945; 346.0877; 345.0727; 333.0790; 325.0946; 302.1002; 275.0803; 267.0761; 253.0587; 249.0576; 240.0484; 226.0711; 198.0746; 171.0566; 121.0387; 115.9012
m_127	19.34	C28H22O6	454.14164	453.13436	453.13515	1.73	[M - H]-	trans-epsilon-viniferin [a]	204. 228. 324	359.0865; 347.0872; 225.0523
m_129	20.62	C28H22O6	454.14164	453.13436	453.13497	1.34	[M - H]-	trans-delta-viniferin [a]	202. 222. 310	385.1347; 369.1079; 359.0865; 347.0872; 333.0713
m_150	19.91	C28H22O7	470.13656	469.12928	469.12539	8.30	[M - H]-	stilbene dimer 469 [b]	208. 240. 312	427.1228; 411.0859; 359.0885; 358.4240; 347.0922; 345.0737; 328.0720; 317.0813; 289.0957; 265.0578; 241.0413; 93.0416
m_152	18.34	C28H22O7	470.13656	469.12928	469.12529	8.51	[M - H]-	scirpusin A isomer 1 [b]	200. 218. 316	451.1167; 375.1119; 359.0947; 347.0972; 346.0844; 333.0700; 265.0587; 251.0392
m_153	18.53	C28H22O7	470.13656	469.12928	469.12891	0.79	[M - H]-	scirpusin A isomer 2 [b]	200. 216. 316	451.1167; 385.1137; 375.1119; 359.0925; 357.0770; 347.0943; 346.3863; 345.0795; 341.0847; 333.0770; 317.0858; 315.1009; 305.0935; 265.0608; 253.0602; 241.0539; 109.0285
m_154	16.07	NA	NA	NA	471.14776		[M - H]-	stilbene dimer 471 [b]	202. 226. 284. 320	453.1379; 452.1147; 346.0742; 93.0435
m_156	14.15	C28H24O7	472.15221	471.14493	471.14418	1.69	[M - H]-	Leachianol F [a]	196. 218	377.1043; 349.0979; 255.0857; 121.0386
m_157	13.99	C28H24O7	472.15221	471.14493	471.14371	2.59	[M - H]-	Leachianol G [a]	198. 232. 280	377.1014; 349.1410; 255.0857; 121.0374
m_158	14.45	NA	NA	NA	471.14874		[M - H]-	restrytisol A [b]	196. 217. 276	377.1047; 349.0982; 255.0857; 121.0384 @ 10 eV
m_178	16.64	NA	NA	NA	521.12573		[M - H]-	m_178 [c]	202. 228. 286	475.1426; 453.1380; 381.0794; 359.1398; 265.0587

m_181	20.62	NA	NA	NA	521.12619		[M - H]-	m_181 [c]	204. 228. 322. 386	476.1416; 475.0926; 474.3192; 453.1393; 433.1099
m_235	19.19	C42H30O9	678.18899	677.18171	677.18142	0.43	[M - H]-	alpha-viniferin [b]	co-eluted peak	583.1400; 571.1392; 570.3190; 529.1274; 501.1475; 500.3037; 465.1122; 464.2970; 463.0805; 437.1036; 436.3068
m_236	19.24	C42H32O9	680.20464	679.19736	679.19738	0.03	[M - H]-	E-miyabenol C isomer-1 [b]	198. 218. 285. 323	661.2191. 637.1855. 585.1508. 555.1469. 573.1399. 451.1163. 438.1003; 357.0827. 345.0803. 335.0960. 227.0788
m_239	19.73	C42H32O9	680.20464	679.19736	679.19782	0.68	[M - H]-	Z-miyabenol C [b]	198. 218. 285	661.2191. 573.1391. 555.1469. 451.1187. 585.1512.345.0793.335.0935.637.1859. 357.0752.227.0773. 467.1107
m_241	18.85	C42H32O9	680.20464	679.19736	679.19822	1.27	[M - H]-	E-miyabenol C isomer-2 [b]	202. 222. 296. 324	661.1602. 637.1876. 585.1521. 573.1391. 451.1210. 357.0784. 345.0784.335.0962
m_242	17.89	C42H32O9	680.20464	679.19736	679.19979	3.57	[M - H]-	ampelopsin C [b]	200. 218. 283	680.2053; 679.1840; 585.1516; 491.0997; 479.1448; 397.0713; 385.0685; 384.0606
m_244	20.81	C42H32O9	680.20464	679.19736	679.19879	2.10	[M - H]-	resveratrol-trimer [b]	co-eluted peak	585.1546; 573.1393; 451.1186; 359.0882
m_264	19.19	NA	NA	NA	745.17190		[M + FA - H]-	m_264 [c]	204. 228. 326	700.1786; 699.1456; 593.1177; 592.1064; 459.1054; 457.0785 @ 40 eV
m_267	18.85	NA	NA	NA	747.18745		[M + FA - H]-	m_267 [c]	co-eluted peak	701.1816; 679.1826; 644.5191
m_293	17.87	C56H42O12	906.26763	905.26035	905.26253	2.15	[M - H]-	hopeaphenol [b]	200. 220. 282	887.2660. 811.2238. 799.2277. 781.2122. 557.1867. 545.1575. 451.1166. 439.1184. 359.0920. 347.934. 333.0795 @ 40 eV
m_300	20.2	C56H42O12	906.26763	905.26035	905.26229	2.14	[M - H]-	vitisin C [b]	206. 226. 286. 328	811.2227. 717.1798. 705.1777. 451.1191. 611.1172. 197.0783. 583.1358. 595.1481 @ 40 eV

m_316	17.8	NA	NA	NA	951.26718	[M - H]$^-$	m_316 [c]	200. 220. 300	907.2843; 906.3020; 905.1635; 904.2006; 679.1873; 678.2596; 485.1798; 475.1424; 474.3192; 452.3338; 248.3816; 244.4271; 180.9889; 174.9725; 151.0406; 150.6052; 119.0434; 113.0101; 112.9857

[1)] All the MS/MS fragment were provided by MS/MS at 25eV expect mentioned

Table S5: SIM method used for 1/20th diluted samples

	Start time (min)	End time (min)	Target ions (m/z)
1	6.08	8.00	103. 117 and 205
2	8.00	10.70	224. 135. 165. 233. 133 and 101
3	10.70	13.50	292. 189. 219. 217. 103. 205. 273. 375.20 and 319.20
4	13.50	14.50	307. 217. 133. 205. 117. 105. 319. 160 and 103
5	14.50	17.65	305. 318 and 191
6	17.65	22.13	361. 437. 129. 204 and 217
7	22.13	24.33	361. 216.95 and 437.20

A filament cut-off has also been implemented	
OFF	ON
5.00	5.50
6.70	7.05

Table S6: SIM method used for non-diluted samples

	Start time (min)	End time (min)	Target ions (m/z)
1	3.26	4.50	116. 100 and 190
2	4.50	6.50	244. 218 and 79

3	6.50	8.90	142. 100. 133. 174. 248. 247. 129. 172. 189. 292. 103. 204. 218. 245. 115. 117 and 101
4	8.90	11.00	174. 248. 290. 224. 135. 165. 128. 243. 115. 232. 100. 218. 117. 103. 219. 304. 156. 230. 258. 292. 220. 205 and 185
5	11.00	13.75	218. 349. 192. 116. 231. 188. 217. 205. 147. 133. 232. 156. 245. 128. 257. 204. 255. 273. 370. 355. 322. 257. 157 and 142
6	13.75	15.50	292. 103. 217. 333. 305. 318. 190 and 292.15
7	15.50	19.00	243. 129 and 205
8	19.00	23.50	361. 204. 217. 368. 355 267. 179. 276. 191. 207. 135 and 401
9	23.50	25.25	361. 204 and 217

A filament cut-off has also been implemented	
OFF	ON
5.00	5.50
6.70	7.05
9.85	9.98
11.54	11.62
13.74	13.87
14.00	14.23
19.62	19.73

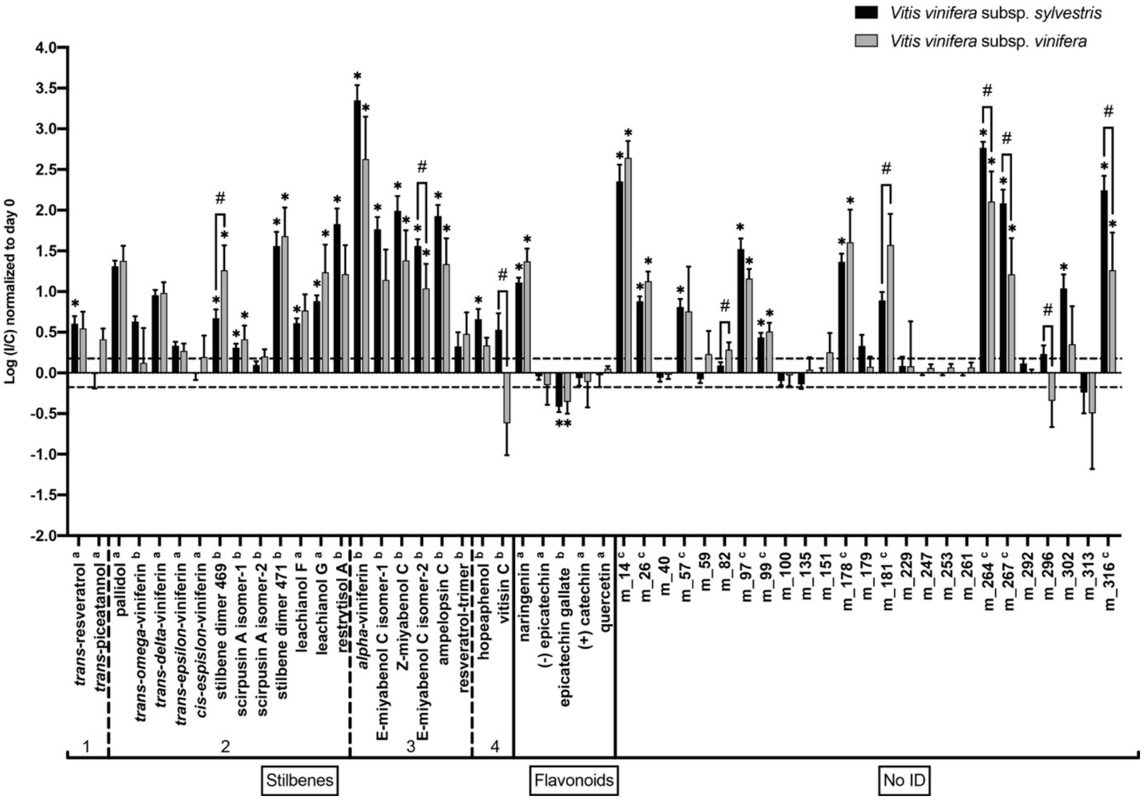

Figure S1. Changes in specialized metabolite contents at three DPI. Contents are expressed as the log of the ratio between content (normalized to T0) in inoculated sample (I) and content in mock-inoculated control sample (C). Metabolite contents are represented in black for *V. v.* subsp. *sylvestris* and in gray for *V. v.* subsp. *vinifera*. * means a statistically significant difference between I and C (Fold Change at least +/- 1.5 and Wilcoxon test, $p<0.05$). # represents a (I/C) statistically significantly different between *V. v.* subsp. *sylvestris* and *V. v.* subsp. *vinifera* ($p<0.05$). [a] indicates metabolites confirmed by standard, [b] indicates putative metabolites identified by MS/MS, and [c] indicates non-characterized metabolites. For stilbenes, 1-2-3, and 4 indicates monomeric, dimeric, trimeric and tetrameric forms respectively.

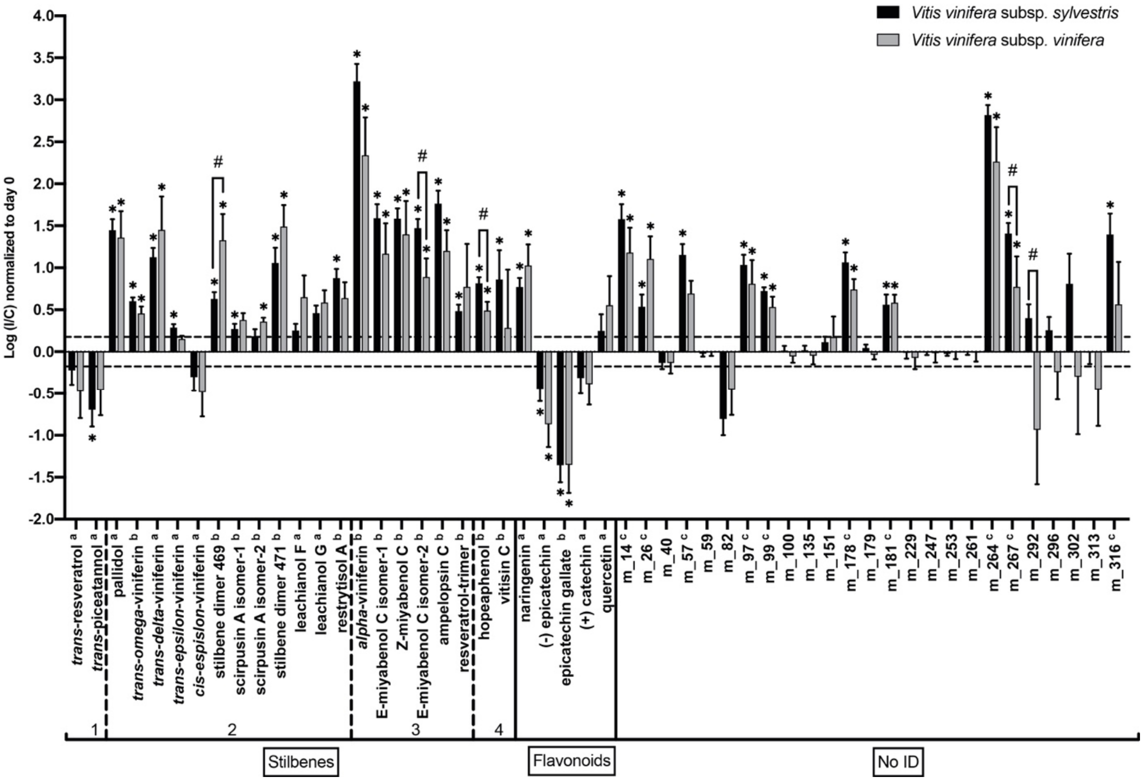

Figure S2. Changes in specialized metabolite contents at seven DPI. Contents are expressed as the log of the ratio between content (normalized to T0) in inoculated sample (I) and content in mock-inoculated control sample (C). Metabolite contents are represented in black for *V. v.* subsp. *sylvestris* and in gray for *V. v.* subsp. *vinifera*. * means a statistically significant difference between I and C (Fold Change at least +/- 1.5 and Wilcoxon test, $p < 0.05$). # represents a (I/C) statistically significantly different between *V. v.* subsp. *sylvestris* and *V. v.* subsp. *vinifera* ($p < 0.05$). [a] indicates metabolites confirmed by standard, [b] indicates putative metabolites identified by MS/MS, and [c] indicates non-characterized metabolites. For stilbenes, 1-2-3, and 4 indicates monomeric, dimeric, trimeric and tetrameric forms, respectively.
